# DNA polymorphisms in inflammatory and endocrine signals linked to frailty are also associated with obesity: data from the FRASNET cohort

**DOI:** 10.3389/fendo.2024.1412160

**Published:** 2024-10-11

**Authors:** Sarah Damanti, Lorena Citterio, Laura Zagato, Elena Brioni, Cristiano Magnaghi, Marco Simonini, Rebecca De Lorenzo, Mariapia Ruggiero, Simona Santoro, Eleonora Senini, Marco Messina, Giordano Vitali, Paolo Manunta, Angelo Andrea Manfredi, Chiara Lanzani, Patrizia Rovere Querini

**Affiliations:** ^1^ Internal Medicine Unit, Istituto di Ricovero e Cura a Carattere Scientifico (IRCCS) San Raffaele Scientific Institute, Milan, Italy; ^2^ Vita-Salute San Raffaele University, Milan, Italy; ^3^ Nephrology and Dialysis Unit, Istituto di Ricovero e Cura a Carattere Scientifico (IRCCS) San Raffaele Scientific Institute, Milan, Italy; ^4^ Scientific Technical Secretariat of the Ethics Committee, Istituto di Ricovero e Cura a Carattere Scientifico (IRCCS) San Raffaele Scientific Institute, Milan, Italy; ^5^ Department of Immunology, Transplantation and Infectious Diseases, Istituto di Ricovero e Cura a Carattere Scientifico (IRCCS) San Raffaele Scientific Institute, Milan, Italy

**Keywords:** obesity, frailty, SNP, predisposition, older people

## Abstract

**Background:**

Obesity and frailty are prevalent geriatric conditions that share some pathophysiological mechanisms and are associated with adverse clinical outcomes. The relationship between frailty, obesity, and polymorphism remains inadequately explored. Single nucleotide polymorphisms (SNPs) offer insights into genetic predispositions that may influence the development of both frailty and obesity.

**Methods:**

We aimed at investigating whether SNPs associated with frailty also play a role in obesity. Data were collected from the FRASNET cross-sectional study, which included community-dwelling older individuals residing in Milan and nearby areas. Participants were recruited through random sampling. They underwent multidimensional geriatric assessments, which included the collection of blood samples for SNP analysis. Frailty was assessed using the frailty index, and body composition was evaluated using bioelectrical impedance analysis and anthropometric measures.

**Results:**

SNPs related to frailty and linked to the renin–angiotensin system (CYP11B2 rs1799998, AGT rs5051, and AGTR1 rs2131127), apoptosis pathways (CASP8 rs6747918), growth hormone signaling (GHR rs6180), inflammation (TLR4 rs5030717, CD33 rs3865444, and FN1 rs7567647), adducin (ADD3 rs3731566), and the 9p21–23 region (rs518054) were found to be associated with various measures of obesity in community-dwelling older adults.

**Conclusions:**

Frailty-related SNPs contribute to obesity in community-dwelling older adults. We identified a novel association between adducin SNPs and visceral fat, which has not been previously reported. Detecting genetic predispositions to obesity and frailty early could aid in identifying individuals at risk, facilitating the adoption of preventive interventions. This represents an initial step toward promoting early intervention strategies.

## 1 Background

The association between frailty and obesity has been documented in numerous cross-sectional and longitudinal studies (1, 2). Obesity and frailty share common pathophysiological mechanisms that collectively worsen the age-related decline in physiological reserves. Key mechanisms include inflammation, oxidative stress, activation of apoptotic pathways, and dysregulation of endocrine axes, all of which are altered in both conditions (3, 4). Abdominal obesity, in particular, significantly contributes to establishing the connection between obesity and frailty (5–9).

The increase in visceral fat leads to a simultaneous infiltration of adipose tissue by inflammatory cells that secrete pro-inflammatory cytokines. This excess of inflammatory cytokines not only exacerbates the phenomenon of “inflammaging” but also contributes to the development of various diseases, thereby increasing frailty (10–12). Hubbard et al. (7) demonstrated that individuals with elevated waist circumference had higher frailty index (FI) and frailty phenotype scores compared to those with normal waist circumference, irrespective of their body mass index (BMI). Additionally, abdominal obesity contributes to the development of metabolic dysfunction, which underpins many cardiovascular comorbidities associated with obesity and frailty. This metabolic dysfunction has also been independently linked to an increased risk of frailty progression, regardless of BMI (13).

However, it is important to note that not all obese individuals are frail, and conversely, not all frail individuals are obese. Given the global epidemics of obesity (14) and frailty in the aging world population (15), it is crucial to identify the factors that confer a genetic predisposition to both conditions. This knowledge could enable early screening and implementation of preventive strategies, potentially mitigating the onset and progression of these serious conditions before they manifest.

The relationship between frailty, obesity, and polymorphism remains inadequately explored. Single nucleotide polymorphisms (SNPs) offer insights into genetic predispositions that may influence the development of both frailty and obesity. These polymorphisms can impact diverse biological pathways involved in inflammatory responses, metabolic processes, and endocrine functions, all of which contribute to these conditions. Understanding these genetic links could illuminate why certain individuals are more susceptible to frailty and obesity, even when exposed to similar lifestyle and environmental factors.

In this observational study, we leveraged data from the FRASNET study to investigate whether frailty-related SNPs also play a role in obesity. Our aims were threefold: i) to identify SNPs that are shared between frailty and obesity; ii) to lay the groundwork for developing genetic screening tools aimed at identifying individuals at risk for both conditions; and iii) to provide insights that could guide early prevention and intervention strategies to mitigate the impact of frailty and obesity in the aging population (see **Table 1**).

**Table 1:** Description of study’s objectives and analyses approaches.

Objective	Description	Analysis approach
1. **Identify Specific SNPs**	Determine which single nucleotide polymorphisms (SNPs) are common between frailty and obesity	Genetic association analysis to identify SNPs linked to both conditions
2. **Develop Screening Tools**	Provide a basis for developing genetic screening tools to identify individuals at risk for both conditions	The SNPs identified as associated with frailty and obesity could be used to predict the development of frailty and obesity in spite of lifestyle and environmental factors
3. **Inform Prevention Strategies**	Offer insights that could inform early prevention and intervention strategies	Correlate genetic findings with clinical data to suggest potential prevention and intervention strategies

By elucidating the relationship between frailty, obesity, and genetic polymorphism, this study aims to provide valuable insights that can enhance health outcomes for the aging population.

## 2 Methods

### 2.1 Study participants

The FRASNET study was a cross-sectional multicenter observational cohort study involving healthy older volunteers (16). Initially, the project aimed to analyze genetic and biochemical factors influencing inflammaging’s effects on kidneys, muscles, cognition, and mood. Additionally, it sought to elucidate genetic and immunological mechanisms contributing to frailty development.

Approval for the study was obtained from the local review board on 9 March 2017, under Protocol No. 24/INT/2017. Healthy volunteers aged 65 years and older were recruited through random sampling methods. Recruitment efforts were conducted at San Raffaele Hospital, Residential Care Facilities for the Elderly and recreational and cultural centers.

Participants provided written informed consent before participating in the study. Enrolment occurred between 1 April 2017 and 16 October 2020, at recreational and cultural centers and retirement homes in Milan and the Monza Brianza areas, Italy. Evaluation visits were conducted at the San Raffaele Scientific Institute in Milan and Cuggiono Hospital near Milan, Italy.

### 2.2 Inclusion/exclusion criteria

The inclusion criteria for participants in the study were individuals aged 65 years or older, capable of walking more than 500 m without assistance, and with a life expectancy exceeding 6 months. Exclusion criteria included severe cognitive impairment [defined as Mini-Mental State Examination (MMSE) score < 18/30], inability to provide written informed consent, and severe health conditions such as uncontrolled hypertension, recent upper or lower extremity fractures, or myocardial infarction within the past year. Institutionalized participants, those lacking data necessary for calculating the FI, and participants with missing data on body composition were excluded from the present analysis. All individuals included in the study signed a written consent, and there were no cases of individuals who did not sign the consent but were willing to participate or cases that declined involvement in the study.

### 2.3 Procedures

Participants underwent comprehensive geriatric assessments, which included the collection of demographic and psychosocial data via a self-administered questionnaire. These assessments covered evaluations of comorbidities, pharmacological therapies, incidence of falls, and emergency department visits within the past year. Anthropometric measurements, cognitive function assessments, mood evaluations, fatigue assessments, quality of life evaluations, and assessments of physical activity were also conducted.

Body composition was determined using the Full Body Sensor Body Composition Monitor and Scale by OMRON (17). Obesity was defined as a fat mass percentage ≥42% in women and ≥30% in men (17). Furthermore, we assessed visceral fat percentage and waist circumference to evaluate abdominal obesity. Muscle performance was evaluated using the Short Physical Performance Battery (SPPB) (18), which includes subtests for standing balance, usual gait speed, and chair-stand test. Muscle strength specifically was measured using the chair-stand subtest, where values >15 s indicated reduced muscle strength and pre-sarcopenia (19).

Blood samples taken were bio-banked and subsequently processed for genetic polymorphism analysis associated with frailty and obesity. Frailty was assessed using a 49-item FI based on criteria defined by Theou et al. (20). The FI encompassed deficits from multidimensional geriatric evaluations, covering i) chronic conditions (e.g., hypertension, cancer, stroke, heart diseases, dyslipidemia, diabetes, psychiatric diseases, and polypharmacy), ii) mental health [assessed using questions from the Short Form Health Survey 36 (SF-36), the Geriatric Depression Scale 15 items (GDS-15), and the Mini Mental State Examination (MMSE)], iii) self-rated health (questions from the SF-36), iv) physical performance (questions from the SF-36, reduction of gait speed, and impaired balance tasks from the SPPB), v) living behavior and social function [questions from the SF-36 and The Physical Activity Scale for the Elderly (PASE) Questionnaire], and vi) signs and symptoms (mean systolic or diastolic blood pressure elevated on three measurements and mean heart rate elevated on three measurements). Each deficit was scored as 0 for absence and 1 for presence, resulting in a score ranging from 0 to 1. **Supplementary Table S1** describes the deficits included in the FI computation. A cutoff point of ≥0.25 defined “frail” individuals. Participants with over 20% missing variables were excluded from the FI computation.

In this study, we performed a targeted gene analysis focusing on SNPs known to be related with frailty (referenced in **Supplementary Table S2**) to explore their role in the association between frailty and obesity. Genomic DNA was extracted from peripheral whole blood using the automated Maxwell^®^ RSC Blood DNA Kit (Promega, Madison, WI, USA). The genetic characterization of SNPs was carried out using the TaqMan OpenArray Genotyping System (Applied Biosystems, Foster City, CA, USA). This state-of-the-art genotyping platform facilitates high-throughput analysis of genetic variations with exceptional accuracy and efficiency. Custom OpenArray plates, designed specifically to accommodate multiple SNPs relevant to our study, were utilized. These plates come pre-loaded with specific assays for the SNPs of interest, enabling a streamlined and automated workflow. The system employs fluorescently labeled probes that bind specifically to DNA sequences of interest, allowing for precise detection and quantification of the target SNPs. This methodology ensures robust and reproducible genotyping results, which are critical for conducting accurate genetic association analyses in our research on frailty and obesity (21).

### 2.4 Statistical analyses

We employed descriptive statistics to summarize the baseline characteristics of the study population. Continuous variables were presented as mean and standard deviation (SD) for normally distributed data or as median and interquartile range (IQR) for skewed distributions. Dichotomous variables were reported as counts (N) and percentages (%). To evaluate the association between genetic polymorphisms known to be related with frailty and various measures of obesity (fat mass percentage, visceral fat percentage, waist circumference, and BMI), we conducted age- and gender-adjusted stepwise linear regression analyses. To control the false discovery rate at α = 5%, we applied the Benjamini–Hochberg procedure. This statistical method helps mitigate the risk of identifying false-positive associations when conducting multiple comparisons in genetic association studies. All statistical analyses were performed with SPSS version 25.0 (SPSS Inc., Chicago, IL, USA).

## 3 Results

In this study, we analyzed data from 1,114 individuals previously enrolled in the FRASNET cohort to investigate whether frailty-related SNPs also contribute to obesity. **Table 2** summarizes the main characteristics of the study population. The median age of participants was 72 years, with 674 (60.5%) women. A majority of the participants were married (70.7%), had a good economic status (>10,000 euro/year; 89.8%), and a high education level (43.8% high school, 18% university).

**Table 2:** Main characteristics of the study population.

	All (N = 1114)
Age	72 (IQR, 69–77)
Male/female	440 (39.5%)/674 (60.5%)
Marital status	
*Married*	788 (70.7%)
*Widower/divorced*	270 (24.2%)
*Single*	56 (5%)
Smoke	382 (34.3%)
Economic status	
*<10,000 euros/year*	97 (8.7%)
*>10,000 euros/year*	1,000 (89.8%)
Education	
*Primary school*	158 (14.2%)
*Secondary school*	265 (23.8%)
*High school*	488 (43.8%)
*University*	200 (18%)
Weight (kg)	69.5 (IQR 61.6–78.7)
Height (cm)	1.61 (IQR 1.55–1.69)
Waist circumference (cm)	92 (IQR 85–101)
BMI (kg/m^2^)	26.8 (IQR 24–29.4)
Fat mass (%)	33.0 (IQR 26.2–40.1)
Visceral fat (%)	11 (IQR 8–14)
Skeletal muscle mass (%)	27.9 (IQR 24.9–31.5)
Short Physical Performance Battery (SPPB)	11 (IQR 9–11)
Gait speed (m/s)	1.16 (IQR 1.0–1.31)
PASE	102 (IQR 65–152)
Chair test (s)	13.0 (IQR 11.0–15.7)
MMSE	27 (IQR 26–30)
Fatigue Severity Scale	26 (IQR 16.5–36)
GDS-15	2 (IQR 0–4)
Hypertension	670 (60.1%)
Diabetes	109 (9.8%)
Dyslipidemia	155 (13.9%)
Chronic Kidney Disease (i.e., GFR < 60 ml/min)	221 (19.8%)
Cardiovascular incidences	217 (19.5%)
Previous stroke	56 (5%)
Psychiatric incidences	101 (9.1%)
FI	0.11 (IQR 0.07–0.20)
FI ≥ 0.25	234 (21%)
Any fall in the year previous the evaluation	243 (21.8%)
ED accesses in the year previous the evaluation	243 (21.8%)
Number of chronic drugs	3 (IQR 1–4)
Polypharmacy	276 (24.8%)

The median FI score was 0.11 (IQR, 0.07–0.20), with 234 individuals (21%) classified as frail (FI ≥ 0.25). Common comorbidities included hypertension (60%), diabetes (9.1%), dyslipidemia (13.9%), chronic kidney disease (19.8%), cardiovascular events (19.5%), and previous stroke (5%). The median number of chronic medications was 3, and polypharmacy prevalence was 24.8%.

Median BMI was 26.8 kg/m^2^ (IQR, 24–29.4), and median waist circumference was 92 cm (IQR, 85–101). Body composition analysis revealed a median skeletal muscle mass of 27.9% (IQR, 24.9–31.5), median fat mass of 33% (IQR, 26.2–40.1), and median visceral fat of 11% (IQR, 8–14%). Median levels of physical activity (PASE score = 102), cognitive performance (MMSE score = 27), and affective status (GDS = 2) were within normal ranges.

**Figure 1:**
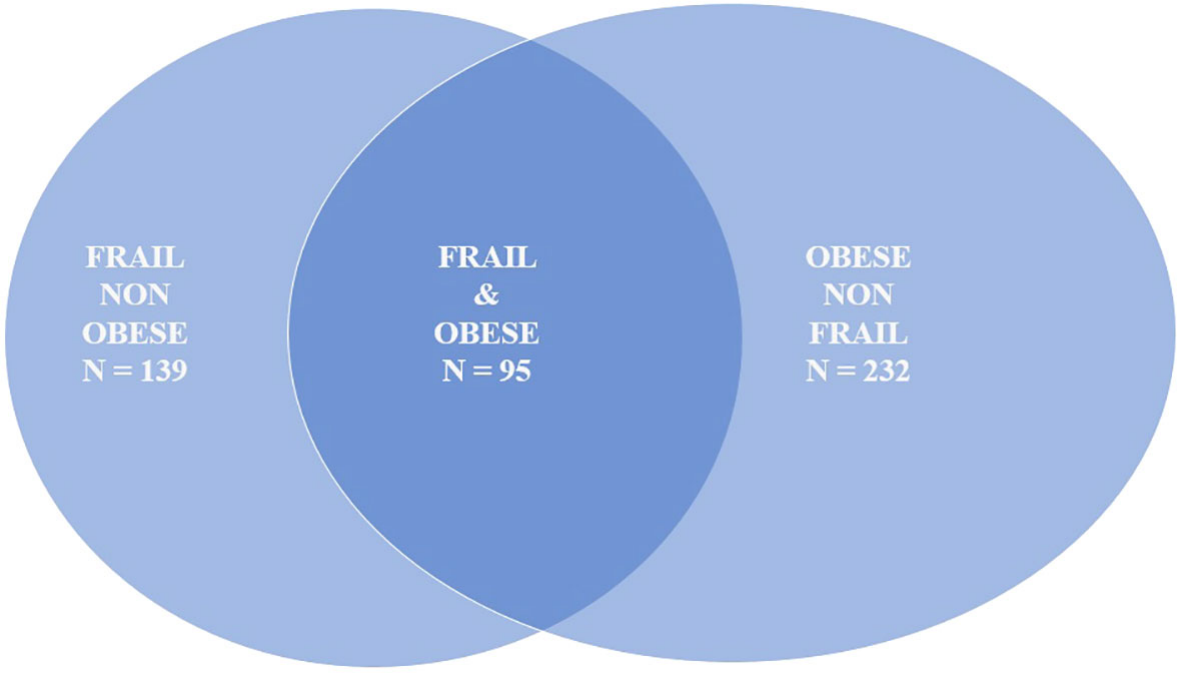
Frailty and obesity overlap in the FRASNET cohort.

In our sample, 327 individuals (29.4%) were obese, with 95 participants classified as both obese and frail (**Figure 1**). **Table 3** presents the associations between frailty-related SNPs and different measures of obesity. Specifically, SNPs in Fibronectin 1 (FN1 rs7567647: B 0.99; 95% CI, 0.17–1.82), Caspase 8 (CASP8 rs6747918: B 1.003; 95% CI, 0.10–1.90), and Adducin 3 (ADD3 rs3731566: B 0.93; 95% CI, 0.15–1.71) genes were associated with visceral fat. Fibronectin 1 (FN1 rs7567647: B 1.54; 95% CI, 0.24–2.85), Caspase 8 (CASP8 rs6747918: B 1.80; 95% CI, 0.37–3.22), rs518054 (GT B 1.72; 95% CI, 0.41–3.04; GG: B 3.19; 95% CI, 0.27–6.12), and Aldosterone Synthase (CYP11B2 rs1799998: B–1.65; 95% CI, −3.02 to −0.27) SNPs were associated with fat mass. Additionally, Angiotensinogen (AGT rs5051: B 2.47; 95% CI, 0.30–4.64), Angiotensin II Receptor Type 1 (AGTR1 rs2131127: B −4.07; 95% CI, −7.44 to −0.70), Toll Like receptor 4 (TLR4 rs5030717: B −14.02; 95% CI, −25.05 to −2.99), Growth Hormone Receptor (GHR rs6180: B 2.68; 95% CI, 0.20–5.15), and CD33 Molecule (CD33 rs3865444: B −2.49; 95% CI, −4.63 to −0.34) SNPs were associated with waist circumference.

**Table 3:** Frailty-related polymorphisms associated with different measures of obesity.

Visceral fat %				
	B	95% C.I.	p	p*
FN1rs7567647_AG	0.99	0.17–1.82	0.02	0.03
ADD3rs3731566_AG	0.93	0.15–1.71	0.02	0.024
CASP8rs6747918_GG	1.003	0.10–1.90	0.029	0.03
Fat mass %				
	B	95% C.I.	p	
CASP8rs6747918_GG	1.80	0.37–3.22	0.01	0.027
rs518054_GT	1.72	0.41–3.04	0.01	0.032
FN1rs7567647_AG	1.54	0.24–2.85	0.02	0.025
CYP11B2rs1799998_TT	−1.65	−3.02–−0.27	0.02	0.03
rs518054_GG	3.19	0.27–6.12	0.03	0.033
BMI				
	B	95% C.I.	p	
FN1rs7567647_AG	1.20	0.37–2.03	0.01	0.06
AGTrs5051_CT	0.98	0.19–1.77	0.02	0.07
rs518054_GT	0.89	0.06–1.71	0.04	0.06
CASP8rs6747918_GG	0.92	0.01–1.83	0.05	0.05
Waist circumference				
	B	95% C.I.	p	
AGTR1rs2131127_TT	−4.07	−7.44–−0.70	0.02	0.042
GHRrs6180_AA	2.68	0.20–5.15	0.03	0.034
TLR4rs5030717_GG	−14.02	−25.05–−2.99	0.01	0.044
CD33rs3865444_GG	−2.49	−4.63–−0.34	0.02	0.040
AGTrs5051_CT	2.47	0.30–4.64	0.03	0.030

*Corrected with the Benjamini–Hochberg procedure to control the false discovery rate.

## 4 Discussion

In this observational study, we identified associations between frailty-related SNPs and various measures of obesity (visceral fat, fat mass percentage, and waist circumference) among community-dwelling older adults. Specifically, SNPs related to the renin–angiotensin system (RAS) (CYP11B2 rs1799998, AGT rs5051, and AGTR1 rs2131127), apoptosis pathways and cellular stress management (CASP8 rs6747918 and CD33 rs3865444), inflammation (TLR4 rs5030717 and FN1 rs7567647), growth hormone signaling (GHR rs6180), adducin (ADD3 rs3731566), and the 9p21–23 region (rs518054) showed significant associations.

Both obesity and frailty are complex, polygenic conditions that often exhibit clinical overlap (1, 2) and share underlying pathophysiological mechanisms (3). Our study contributes to understanding a shared genetic predisposition that promotes the development of both conditions. Importantly, we have identified, for the first time, an association between the adducin (ADD3 rs3731566) SNP and the rs518054 SNP in the 9p21–23 region with obesity.

The SNPs identified as associated with both frailty and obesity hold potential as biomarkers for predicting the onset of these conditions, independent of lifestyle and environmental influences. Integrating these genetic markers into screening tools could effectively identify individuals with a heightened genetic susceptibility to frailty and obesity from an early age. This proactive identification could facilitate targeted prevention strategies and personalized interventions aimed at reducing the impact of these complex health issues.

The RAS, in addition to its traditional role in regulating electrolyte balance and cardiovascular function, can also be locally synthesized in various tissues (22). Local activation of the RAS has been associated with sarcopenia, a significant factor in physical frailty (22). Furthermore, RAS activity at the mitochondrial level enhances oxidative stress (22, 23), leading to compromised mitochondrial function and integrity. This reduction in ATP production contributes to decreased energy levels, which is a critical component of frailty. Moreover, excessive reactive oxygen species (ROS) from mitochondrial dysfunction can affect hypothalamic neurons involved in regulating appetite (24), potentially influencing obesity.

Apoptosis and impaired cellular stress management are critical biological mechanisms underlying frailty (25). Obesity, similarly, has been associated with a pro-apoptotic phenotype in both animal and human studies (26). Specifically, caspases, which play pivotal roles in the later stages of apoptosis at the mitochondrial level (27), have been shown to be activated in adipocytes of obese mice and humans (27). In our study, we observed an association between the CASP8 SNP and measures of fat mass and visceral fat. CD33, involved in cellular stress management and repair of ROS-induced cell damage (28), showed a negative association with waist circumference in our analysis, suggesting an increased vulnerability to ROS damage among obese individuals.

Inflammation, recognized as one of the six “hallmarks of aging” (29), plays a pivotal role in driving frailty (25) and is closely intertwined with obesity and its metabolic complications. White adipocytes, particularly those found in visceral fat, express monocyte chemoattractant protein (MCP)-1 (30), which attracts monocytes from the bloodstream. These monocytes differentiate into macrophages within adipose tissue, where they secrete pro-inflammatory cytokines (31). Toll-like receptors (TLRs), part of the innate immune system, typically recognize pathogen-associated molecular patterns (PAMPs) rich in lipids (32). However, they can also bind to saturated fatty acids consumed in excess and fibronectin (33), initiating an inflammatory signaling cascade. Previous research has indicated a correlation between TLR4 expression and BMI (34). Our study revealed associations between a TLR4 SNP and visceral fat and between a FN1 SNP and both overall and visceral fat accumulation.

The role of the GH axis in determining frailty and obesity remains a subject of debate. Physiologically, the production of GH and IGF-1 declines with age, contributing to changes in body composition associated with aging (35). Obese individuals typically exhibit lower GH pulsatile secretion compared to those with normal weight (36), and diminished levels of GH and IGF-1 have been linked to an elevated risk of chronic conditions such as diabetes and heart disease (37). Studies by Mohamad et al. demonstrated reduced IGF-1 levels in frail men compared to robust controls (38). Interestingly, lower IGF-1 levels have been associated with increased survival probability in some contexts (39, 40), and mutations in the GH receptor that reduce its activity have been linked to protection against several age-related diseases (41). In our study, we observed an association between the GHR rs6180 AA SNP and increased waist circumference.

Adducin is a membrane skeleton protein crucial for signal transduction in various cellular physiological processes. Genetic variations in adducin can influence these functions, impacting multiple pathogenic pathways. Previous research has associated adducin polymorphisms with conditions such as hypertension, cancer, and biliary atresia (42). However, to our knowledge, no prior study has demonstrated an association between adducin SNPs and visceral fat.

The 9p21–23 region is recognized as a risk locus for various diseases, and its rs518054 SNP has previously been associated with frailty (43). Our study provides novel evidence demonstrating that the rs518054 SNP in this region is also associated with obesity.

Our study has the merit of illustrating the association between various genetic polymorphisms linked to frailty and different measures of obesity in community-dwelling older adults. These findings suggest a common genetic predisposition that influences both conditions. However, some limitations should be mentioned: the regional nature of the study, which may impair the generalizability of our findings, and the cross-sectional design, which prevents us from inferring any cause-effect relationships between the frailty SNPs and obesity. Future research should focus on longitudinal studies and include diverse populations to validate and expand upon these findings.

## 5 Conclusions

By identifying SNPs associated with both frailty and obesity, we have enhanced the knowledge of the genetic determinants underlying these conditions. This may help explain why these prevalent geriatric conditions do not always coincide. Furthermore, early identification of genetic risk factors for obesity and frailty could facilitate the timely implementation of preventive interventions, potentially mitigating the onset of these conditions and their associated adverse outcomes.

## Data Availability

The original contributions presented in the study are publicly available. The data in the San Raffaele Open Research Data Repository, V1 can be found here: 10.17632/wd77gnrmxw.1, and the SNP data can be found here: https://www.ncbi.nlm.nih.gov/SNP/snp_viewBatch.cgi?sbid=1063657.
