# Supplementary material for: DNA polymorphisms in inflammatory and endocrine signals linked to frailty are also associated with obesity: data from the FRASNET cohort
Source: Front Endocrinol (Lausanne). 2024 Oct 11;15:1412160. doi: 10.3389/fendo.2024.1412160 (PMC11502925; doi:10.3389/fendo.2024.1412160)
Supplement: Supplementary file 1 [file Table1.pdf]

Table S1: Variables included in the computation of the frailty index

Hypertension
Active cancer
Previous cancer
Stroke
Heart disease
Dyslipidemia
Diabetes
GFR ≤ 60 ml/min
Psychiatric disease
Politherapy (chronic drugs ≥ 5)
Geriatric Depression Scale 15 items ≥ 5
SPPB balance < 2
Gait speed reduced (≤ 0.8 m/s)
MMSE ≤ 24
Exercise (PASE < 76)
Never walking (PASE_days_never_walking)
Not used to perform social activities
Insomnia
Mean Systolic Blood Pressure elevated
Mean Diastolic Blood Pressure elevated
Mean Heart rate elevated
Abnormal natremia (Na < 135 mmol/l or Na > 145 mmol/l)
Abnormal kaliemia (K < 3.5 mmol/l or K > 5 mmol/l)
Question 1 SF-36: in general your health is fair or scarse (answers 4 or 5)
Question 4 SF-36 (Moderate Physical activities like moving a table or using the hoover or cycling) = 1 (very limited)
Question 5 SF-36 (Carrying the shopping bags) = 1 (very limited)
Question 6 SF-36 (Climbing a few flights of stairs) = 1 (very limited)
Question 8 SF-36 (Bend, kneel or stoop) = 1 (very limited)
Question 9 SF-36 (Walking for 1 km) = 1 (very limited)
Question 12 SF-36 (Bathing or dressing autonomy) = 1 (very limited)
Question 13 SF-36: in the last month reduced time dedicated to work and other activities due to physical problems = 1
Question 14 SF-36: in the last month your performance was reduced due to physical problems = 1
Question 16 SF-36: in the last month difficulties in working or performing other activities due to physical problems = 1
Question 17 SF-36: in the last month reduced time dedicated to work and other activities due to emotional problems = 1
Question 18 SF-36: performance less than expected due to emotional problems = 1
Question 19 SF-36: reduced concentration due to emotional problems = 1
Question 20 SF-36: in the last month your physical or emotional status interfered with the normal social activities with the family and friends = 4 (much) or 5 (very much)
Question 21 SF 36: in the last month physical pain was moderate (4) or intense (5) or very intense (6)
Question 22 SF 36: in the last month pain interfered with working and in house activities much (4) or very much (5)
Question 24 SF 36: in the last month feeling very agitated always (1), almost always (2) or a lot of time (3)
Question 25 SF 36: in the last month feeling almost blue always (1), almost always (2), a lot of time (3)
Question 27 SF36: in the last month did you feel full of energy = almost never (5), never (6)
Question 28 SF 36: in the last month feeling discouraged and sad always (1), almost always (2), a lot of time (3)
Question 29 SF 36: in the last month feeling exhausted always (1), almost always (2) or a lot of time (3)
Question 30 SF 36: in the last month feeling happy never (6) or almost never (5)
Question 31 SF 36: in the last month feeling tired always (1), almost always (2) or a lot of time (3)
Question 32 SF 36: in the last month your physical or emotional status interfered with the normal social activities with the family and friends = 1 (always) or almost always (2)
Question 33 SF-36: I seem to get sick more easily than others = for sure true (1), almost true (2)
Question 36 SF-36: I have an optimal health = almost false (4) or for sure false (5)
