# Supplementary material for: DNA polymorphisms in inflammatory and endocrine signals linked to frailty are also associated with obesity: data from the FRASNET cohort
Source: Front Endocrinol (Lausanne). 2024 Oct 11;15:1412160. doi: 10.3389/fendo.2024.1412160 (PMC11502925; doi:10.3389/fendo.2024.1412160)
Supplement: Supplementary file 2 [file Table2.pdf]

Table S2 SNPs included in the analyses

ADD1rs4961
ADD2rs4984
ADD3rs3731566
AGTrs5051
AGTR1rs2131127
CASP8rs6747918
CD33rs3865444
CYP11B2rs1799998
FN1rs7567647
GHRrs6180
IL6rs1800795
IL10rs1800896
KLrs9536314
rs518054
TLR4rs5030717
